# Efficacy and safety of Water Vapor Thermal Therapy in the treatment of benign prostate hyperplasia: a systematic review and single-arm Meta-analysis

**DOI:** 10.1186/s12894-023-01237-2

**Published:** 2023-04-28

**Authors:** Junyi Yang, Weisong Wu, Yirixiatijiang Amier, Xianmiao Li, Wenlong Wan, Chang Liu, Yucong Zhang, Xiao Yu

**Affiliations:** 1 Department of Urology, Institute of Urology, Tongji Hospital, Tongji Medical College, Huazhong University of Science and Technology, Wuhan, 430030 China; 2 Department of Geriatrics, Institute of Urology, Tongji Hospital, Tongji Medical College, Huazhong University of Science and Technology, Wuhan, 430030 China

**Keywords:** BPH, Rezūm, Water vapor thermal therapy, Systematic review, Meta-analysis

## Abstract

**Background:**

Benign prostatic hyperplasia (BPH) is a common chronic condition among men aged 50 or older, causing voiding and obstructive lower urinary tract symptoms. Water vapor thermal therapy (WVTT) using the Rezūm® system is a new minimally invasive surgical technique that is increasingly reported as a treatment for BPH.

**Methods:**

The protocol was submitted to the PROSPERO registry. We searched PubMed, Web of Science, Embase, Cochrane Library and ClinicalTrials.gov up to July 29, 2022. Quality assessment was carried out by a 20-item checklist form prepared by the Institute of Health Economics (IHE). Double arcsine transformation was performed to stabilize the variance of the original ratio. When I^2^ > 50%, the random effect model was used to calculate the pooled parameters. Otherwise, the fixed effect model was used. 95% confidence intervals (CIs) were calculated. A leave-one-out sensitivity analysis was performed to evaluate the impact of each study on the pooled outcomes, and finally, Egger’s test was used to assess publication bias.

**Results:**

A total of seven single-arm observational studies and one random controlled trial, including 1015 patients, were included. One year after WVTT, the International Prostate Symptom Score decreased by 11.37 (95% CI: -12.53, -10.21), the IPSS Quality of Life scale decreased by 2.59 (95% CI: -2.92, -2.26), the maximum urine flow rate increased by 5.26 ml/s (95% CI: 4.53, 5.99), and the postvoid residual decreased by 13.18 ml (95% CI: -24.32, -2.03). The most common complication was dysuria, with a pooled incidence of 21% (95% CI: 14%, 29%), and the second most common complication was hematuria, with a pooled incidence of 14% (95% CI: 10%, 18%). The pooled incidence of retreatment was 3% (95% CI: 2%, 5%).

**Conclusions:**

WVTT is an attractive alternative to medication or more invasive surgical procedures and can serve as first-line therapy for men with BPH.

**Supplementary Information:**

The online version contains supplementary material available at 10.1186/s12894-023-01237-2.

## Introduction

Benign prostatic hyperplasia (BPH) is an enduring condition that affects many men over the age of 50 [1]. An enlarged prostate and increased prostatic smooth muscle tone, which compresses the prostatic urethra, cause urinary leakage and obstructive lower urinary tract symptoms (LUTS) that often require medical intervention [2]. As the population ages, the incidence of symptomatic BPH will increase proportionally with demographic changes, placing extreme pressure on the healthcare system [3].

Although many well-established and well-studied treatments are available, most men are reluctant to undergo surgical treatment because of its potential impact on sexual function [3]. However, many men fail to see satisfactory symptomatic relief over time or experience various adverse effects that lead to discontinuation [4].

In terms of surgical treatment, transurethral resection of the prostate (TURP) is considered the gold standard [5]. However, it can lead to certain postoperative complications, such as ejaculation disorders, erectile dysfunction and urethral strictures [6]. In recent years, minimally invasive surgical techniques (MISTs) have been developed, such as transurethral microwave thermotherapy and radiofrequency (RF)-induced transurethral needle ablation [7]. However, the inadequate durability and high retreatment rates of these modalities hinder their widespread adoption [8]. Prostatic urethral lift (UroLift), another minimally invasive surgical technique, has been recommended by the UK National Institute for Health and Care Excellence (NICE) for the treatment of LUTS. However, its effectiveness in treating large prostate volumes and obstructive middle lobes remains unclear [9].

Water vapor thermal therapy (WVTT) by the Rezūm^®^ system (Boston Scientific, NxThera, Inc., Maple Grove, MN, USA) is a new MIST. With the use of RF current, this novel device generates convective water vapor energy that passes through tissue interstices and damages cell membranes to cause fast cell death and necrosis [7]. The natural barriers between the prostate zones prevent water vapor dispersal, limiting it to the transition zone [10]. As a result, nearby regions such as the peripheral zone, striated urinary sphincter, bladder, or rectum are spared from thermal damage [6]. Other advantages of WVTT include the fact that it can be performed in the office or as an outpatient procedure under intravenous sedation and prostate block [11]. It minimizes the costs associated with hospitalization and related complications, which is especially important in the COVID-19 era [6].

Many studies have shown that convective RF thermal therapy with the Rezūm^®^ system results in significant and durable improvements in LUTS while preserving sexual function [3, 4, 11, 12]. However, no relevant systematic analysis has been performed to date. Therefore, we conducted this single-arm meta-analysis to evaluate the efficacy and safety of WVTT in the treatment of BPH.

## Methods

We followed the Preferred Reporting Items for Systematic Reviews and Meta-Analysis (PRISMA) guidelines and prospectively registered our protocol on PROSPERO (Registration number: CRD42022350207).

### Data sources and search strategy

We performed a systematic search of published studies in PubMed, Embase, Web of Science, ClinicalTrials.gov, and the Cochrane Library from inception to July 29, 2022. The search items included medical subject headings (MeSH) and the following keywords: “Prostatic Hyperplasia”, “BPH”, “benign prostatic hyperplasia”, “prostatic adenoma”, “benign prostatic hypertrophy”, “prostatic hypertrophy”, “water vaporvapor thermal therapy”, “WVTT”, and “Rezūm”. These terms were combined using Boolean operators (“AND” or “OR”). Detailed search strategies are provided in Additional File.

### Study selection

We included full texts of randomized controlled trials (RCTs), cohort studies, and case‒control studies that evaluated the efficacy and safety of WVTT in BPH treatment. Only articles written in English were included. We excluded abstracts, case reports, reviews, animal studies, editorials, and letters to editors. Duplicate studies and studies with insufficient data were excluded. Two investigators (JYY and WSW) independently screened and selected the studies based on the search strategies for the final review. Disagreements were resolved by discussion with a third investigator (XY).

### Data extraction

The characteristics of the included studies are summarized as follows: the last name of the first author, publication year, study type, country, follow-up duration, sample size, anaesthesia styles, injection times and postoperative outcomes. Data presented as the median and interquartile range (IQR) were converted to the mean ± standard deviation (SD) using mathematical formulas for meta-analysis [13–15].

### Outcomes

The primary outcomes were efficacy parameters, including the International Prostate Symptom Score (IPSS), IPSS Quality of Life Scale (QoL), postvoid residual (PVR), and maximum urine flow rate (Qmax). Secondary outcomes included the incidence of complications, including hematuria, dysuria, hematospermia, urinary tract infection (UTI), and pelvic pain. The changes in the Benign Prostate Hypertrophy Impact Index (BPH II), the International Index of Erectile Function erectile function domain (IIEF-EF), the Male Sexual Health Questionnaire for Ejaculatory Dysfunction (MSHQ-EjD), and the MSHQ-bother were also evaluated. The effect size was the change in outcomes before and after WVTT.

### Statistical analysis

The meta-analysis was conducted by using Stata 14.0 software. Double arcsine transformation was used for binary data that did not follow the normal distribution to stabilize the variance of the original ratio, and the final results were presented as percentages (proportion × 100) with 95% confidence intervals (95% CI). The outcome of the continuous data was presented as the mean difference (MD) and 95% CI. A P value of < 0.05 was considered statistically significant. The heterogeneity of effect size was assessed using the Q statistic and I^2^ (I^2^ value > 50% indicates high heterogeneity). When I^2^ > 50%, we used a random-effect model to pool effect sizes; otherwise, we used a fixed-effect model. A leave-one-out sensitivity analysis was performed for each of the highly heterogeneous results to identify the source of heterogeneity. If the heterogeneity became acceptable and the p value was stable after excluding a study (I²<50%), we excluded this study and pooled the remaining studies to obtain a more stable result.

### Quality and publication bias assessment

A relatively systematic and comprehensive quality assessment tool developed by the Canadian Institute of Health Economics (IHE) was used for quality assessment [16]. Studies that met 16 or more of the 20 items were considered to be of acceptable quality. Two authors (WLW and XML) independently assessed each study for bias. Disagreements were resolved by consensus. Eventually, we performed Egger’s test to quantitively assess publication bias, with a p value < 0.05 considered statistically significant for publication bias.

## Results

### Search results

**Fig. 1:**
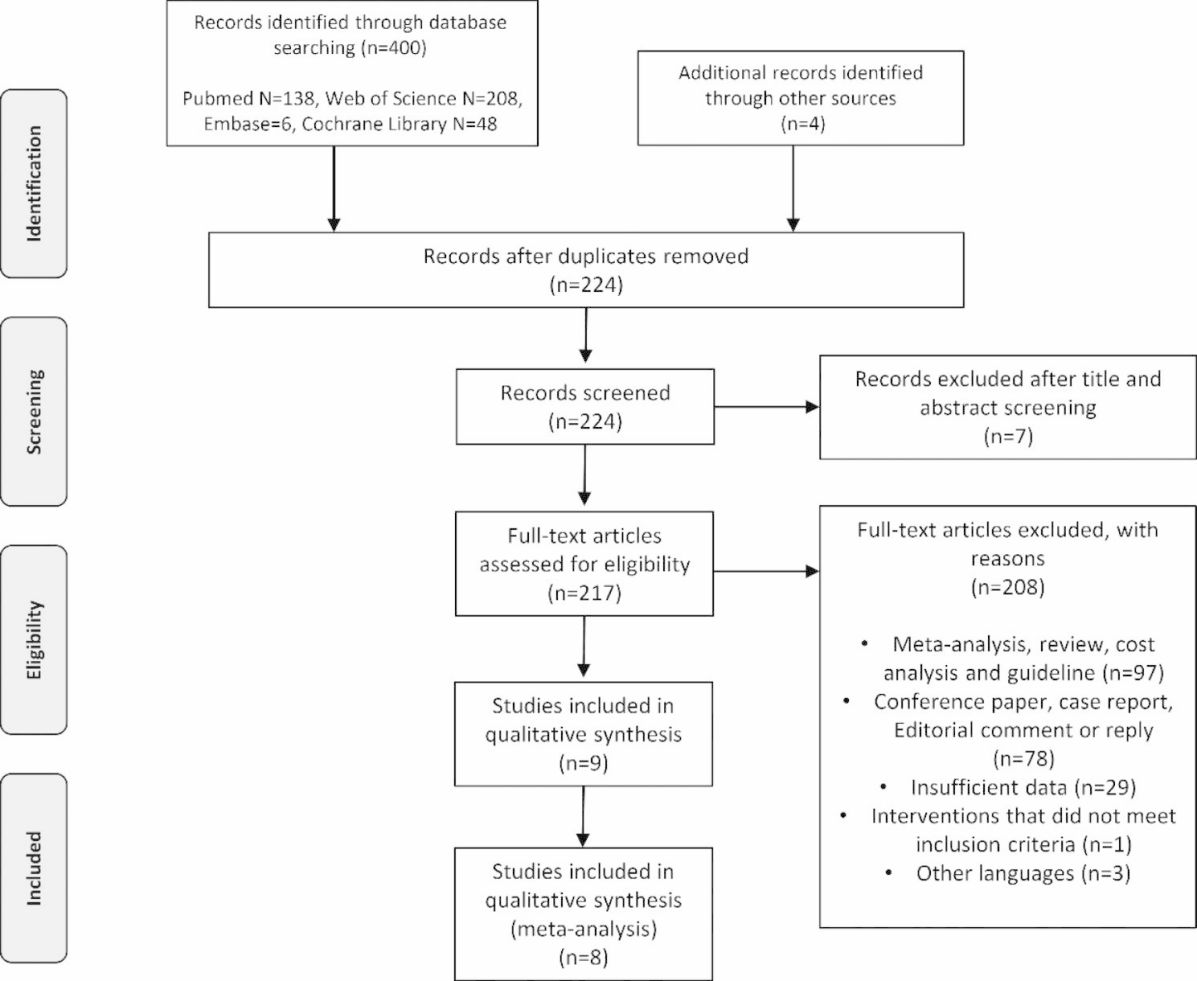
PRISMA flow diagram of the selection process

Our initial search found a total of 404 studies. After preliminary screening and full-text assessment, eight studies were included in the meta-analysis [2–8, 10, 11]. Figure 1 shows the PRISMA flow chart illustrating how the final studies were selected. As shown in Table 1, the quality assessment score of all studies was 16 or higher, except for the study reported by Haroon et al. [17]. Tables 2 and 3 show the baseline characteristics of the eight studies and all patients, respectively. These studies were conducted in different countries, including France, the USA, Dominica, the Czech Republic, Sweden and Canada. The follow-up duration of seven studies was one year or more. A total of 1015 patients were included in these 8 studies. These patients had moderate to severe LUTS secondary to BPH.

**Table 1:** Quality assessment results of the included studies by the IHE case series quality assessment tool

items		study									
		Alegorides	Darson	Dixon	Elterman	Fernández-Guzmán		Haroon	Ines	McVary	Wasserbauer
		2020	2017	2016	2022	2022		2022	2021	2016	2021
**1**	**The hypothesis, purpose and objective of the study clearly stated.**	1	1	1	1	1		1	1	1	1
**2**	**Describe the characteristics of the patient.**	1	1	1	0	1		1	1	1	1
**3**	**Multicenter case collection.**	1	1	1	1	1		0	0	1	0
**4**	**The inclusion and exclusion criteria are clear and reasonable.**	1	0	1	0	1		1	1	1	1
**5**	**Include patients consecutively.**	1	1	1	1	0		1	1	1	1
**6**	**Patients are in the same condition.**	1	1	1	1	1		1	1	1	1
**7**	**Describe the interventions clearly.**	1	1	1	1	1		1	1	1	1
**8**	**Describe joint interventions clearly.**	0	0	0	0	1		0	0	1	0
**9**	**Clarify the outcome of the measurement in advance.**	1	1	1	1	1		1	1	1	1
**10**	**Reasonable objective and/or subjective methods to measure outcome.**	1	1	1	1	1		1	1	1	1
**11**	**Outcome parameters were measured before and after intervention.**	1	1	1	1	1		1	1	1	1
**12**	**Reasonable statistical tests are used to evaluate the result parameters.**	1	1	1	1	1		1	1	1	1
**13**	**Report follow-up time.**	1	1	1	1	1		1	1	1	1
**14**	**Report loss follow-up.**	1	1	1	1	1		0	1	1	1
**15**	**Data analysis of outcome measures provided random variable estimates.**	1	1	1	1	1		1	1	1	1
**16**	**Report intervention-related adverse events.**	1	1	1	1	1		0	1	1	1
**17**	**Research results support conclusions.**	1	1	1	1	1		1	1	1	1
**18**	**Statement of conflicts of interest and sources of support for the research.**	1	1	1	1	1		0	1	1	1
**19**	**Prospective research.**	0	0	1	1	1		1	0	1	1
**20**	**Blind for outcome reviewers.**	1	1	0	0	0		0	1	1	0
	**Score**	**18**	**17**	**18**	**16**	**18**	**14**		**17**	**20**	**17**

**Table 2:** Baseline characteristics of studies

Author, year	Study type	Country	Follow-up duration	Enrollment	Anaesthesia styles (n/%)	Injections times	Efficacy endpoints	Safety endpoints
Alegorides, 2020	Retrospective observational study	France	12 months	62	hypnosis (12/19.4%) and general anaesthesia (50/80.6%)	total injections: median 5 (range 2–11)	Change of IPSS, Qol, PVR, Qmax, MSHQ-EjD, MSHQ-bother in 1, 3, 6, 12 months	Hematuria, Dysuria, Hematospermia, UTI, Urinary retention, Pelvic pain
Darson, 2017	Retrospective observational study	USA	12 months	131	intravenous sedation (76%), general anaesthesia (15%) and prostate block (6%)	lateral lobes: mean 4.4 (range 2–12); median lobes: mean 1.6 (range 1–6)	Change of IPSS, Qol, PVR, Qmax, in 1, 12 months	Urinary retention
Dixon, 2016	Prospective multicenter single-arm study	Dominica, Czech and Sweden	24 months	65	oral sedation (51/78.5%) and intravenous sedation (14/21.5%)	lateral lobes: mean 4.6 (range 2–9); median lobes mean 1.8 (range 1–3)	Change of IPSS, Qol, PVR, Qmax, BPH II, IIEF-EF, MSHQ-EjD, MSHQ-bother in 1, 3, 6, 12, 24 months	Hematuria, Dysuria, UTI, Urinary retention, Urinary urgency, Urinary frequency
Elterman, 2022	Prospective multicenter single-arm study	Canada	12 months	229	intravenous sedation (137/59.8%), prostate block (44/19.2%) and oral sedation (10/7.3%)	total injections: mean 11 (range 4–28)	Change of IPSS, Qol, PVR, Qmax, BPH II, MSHQ-EjD, MSHQ-bother in 1, 3, 6, 12 months	
Fernández-Guzmán, 2022	Prospective multicenter single-arm study	Spain	12 months	137	intravenous sedation (137/100%)	total injections: mean 5.21 (range 2–14)	NA	Hematuria, Dysuria, Hematospermia, UTI, Urinary retention, Urinary urgency, Urinary frequency, Pelvic pain
Ines, 2021	Retrospective observational study	USA	12 months	179	intravenous sedation (156/87.2%) and prostate block (23/12.8%)	30–80 ml PV: lateral lobes: mean 2.2 (SD 0.7); median lobes: 1; <30 ml PV: lateral lobes: mean 2.2 (SD 0.7); median lobes: 1 and > 80 ml PV: lateral lobes: mean 5.5 (SD 1.8); median lobes: 1	Change of IPSS, Qol, BPH II in 1, 3, 6, 12 months and change of PVR, Qmax, IIEF-EF in 3, 6, 12 months	Hematuria, Dysuria, UTI, Urinary retention
McVary, 2016	Rct	USA	5 years	136	intravenous sedation: 20, prostate block: 41 and oral sedation: 135 (total in intervention and control groups)	total injections: mean 4.5 (SD 1.8)	Change of IPSS, Qol, PVR, Qmax in 1, 3, 6, 12, 24 months and change of BPH II, IIEF-EF, MSHQ-EjD, MSHQ-bother in 3, 6, 12, 24 months	Hematuria, Dysuria, Hematospermia, UTI, Urinary retention, Urinary urgency, Urinary frequency, Pelvic pain
Wasserbauer, 2021	Prospective single-arm study	Czech	3 months	76	analgosedation (70/92.1%), prostate block (5/6.6%) and genaral anaesthesia (1/1.3%)	total injections: range 2–3	NA	Hematuria, UTI, Urinary retention, Urinary urgency

Abbreviations: *IPSS: International Prostate Symptom Score, Qol: IPSS quality of life scale, PVR: postvoid residual, Qmax: maximum urine flow rate, BPH II: Benign Prostate Hypertrophy Impact Index, IIEF-EF: International Index of Erectile Function erectile function domain, MSHQ-EjD: Male Sexual Health Questionnaire for Ejaculatory Dysfunction, UTI:urinary tract infection, SD: standard deviation, PV: prostate volume*

**Table 3:** Baseline characteristics of patients

Characteristics	Alegorides,2020	Darson,2017	Dixon,2016	Elterman,2022	Fernández-Guzmán,2022	Ines,2021	Ines^*^,2021	Ines^#^,2021	McVary,2016	Wasserbauer,2021	
No. of patients	62	131	65	229	137	140	26	13	136	76	
Age (years)	64.3 ± 11.9	70.9 ± 9.4	66.6 ± 7.7	67.3	65.31	64.5 ± 8.4	60.4 ± 9.2	67.2 ± 8.2	63 ± 7.1	65.3 ± 7.1	
Median lobe (%)	29 (46.7%)	54 (41.2%)	14 (21.5%)	126 (55%)	64 (46.7%)	100 (55.9%)			42 (30.9%)	NA	
Bladder catheter (%)	8 (12.9%)	NA	NA	16 (7.0%)	NA	NA			NA	6 (7.9%)	
Prostate volume (ml)	54.3 ± 28.4	45.1 ± 23.4	48.6 ± 20.5	71.5	50.38 ± 18.45	47.6 ± 13	25.2 ± 2.8	93.6 ± 9.9	45.8 ± 13	61.8 ± 29.2	
PSA (ng/L)	2.9 ± 2.7	3.5 ± 5.6	3.9 ± 4.2	NA	2.4	2.5 ± 2.3	0.8 ± 0.9	12.2 ± 11.7	2.1 ± 1.5	3.5 ± 2.8	
IPSS	19.9 ± 6.3	19.5 ± 6.6	21.6 ± 5.5	NA	21 ± 4.63	18.7 ± 6.8	21.5 ± 6.9	18.4 ± 5.5	22 ± 4.8	19.1 ± 6.3	
Qol	NA	4.3 ± 1.2	4.3 ± 1.1	NA	4.09 ± 0.93	4.4 ± 1.3	4.8 ± 1.4	4.1 ± 0.9	4.4 ± 1.1	4 ± 1.1	
PVR (ml)	78.9 ± 88.9	216.8 ± 286.6	92.4 ± 77.3	NA	NA	34.9 ± 64.2	18 ± 49.8	30.2 ± 46.8	82 ± 51.5	67.7 ± 98.1	
Qmax (ml/s)	11.0 ± 3.4	8.6 ± 4.9	7.9 ± 3.2	NA	8.98 ± 4.06	9.3 ± 2.8	9.1 ± 2.8	6.2 ± 2.7	9.9 ± 2.3	8.8 ± 3.7	
BPH II	NA	NA	6.8 ± 2.8	NA	NA	NA	NA	NA	6.3 ± 2.8	NA	
IIEF-EF	NA	NA	13.3 ± 12	NA	NA	15.6 ± 8.6	18.6 ± 8.4	14.1 ± 9.3	17.2 ± 10.3	NA	
MSHQ-EjD	8.6 ± 4.9	NA	5.9 ± 4.8	NA	NA	NA	NA	NA	7.8 ± 4.1	NA	
MSHQ-bother	2.0 ± 1.7	NA	2.3 ± 2.3	NA	NA	NA	NA	NA	2.6 ± 1.7	NA	

Note: Data are presented as the mean ± standard deviation (SD) or mean.

^#^ *: The study was divided into three groups based on the prostate volume. with group 1 (Ines,2021) 30–80 ml, group 2 (Ines^*^,2021) < 30 ml and group3 (Ines^#^,2021) > 80 ml.

Abbreviations: *PSA: Prostate specific antigen; IPSS: International Prostate Symptom Score, Qol: IPSS quality of life scale, PVR: postvoid residual, Qmax: maximum urine flow rate, BPH II: Benign Prostate Hypertrophy Impact Index, IIEF-EF: International Index of Erectile Function erectile function domain, MSHQ-EjD: Male Sexual Health Questionnaire for Ejaculatory Dysfunction*

### Pooled outcomes

**Fig. 2:**
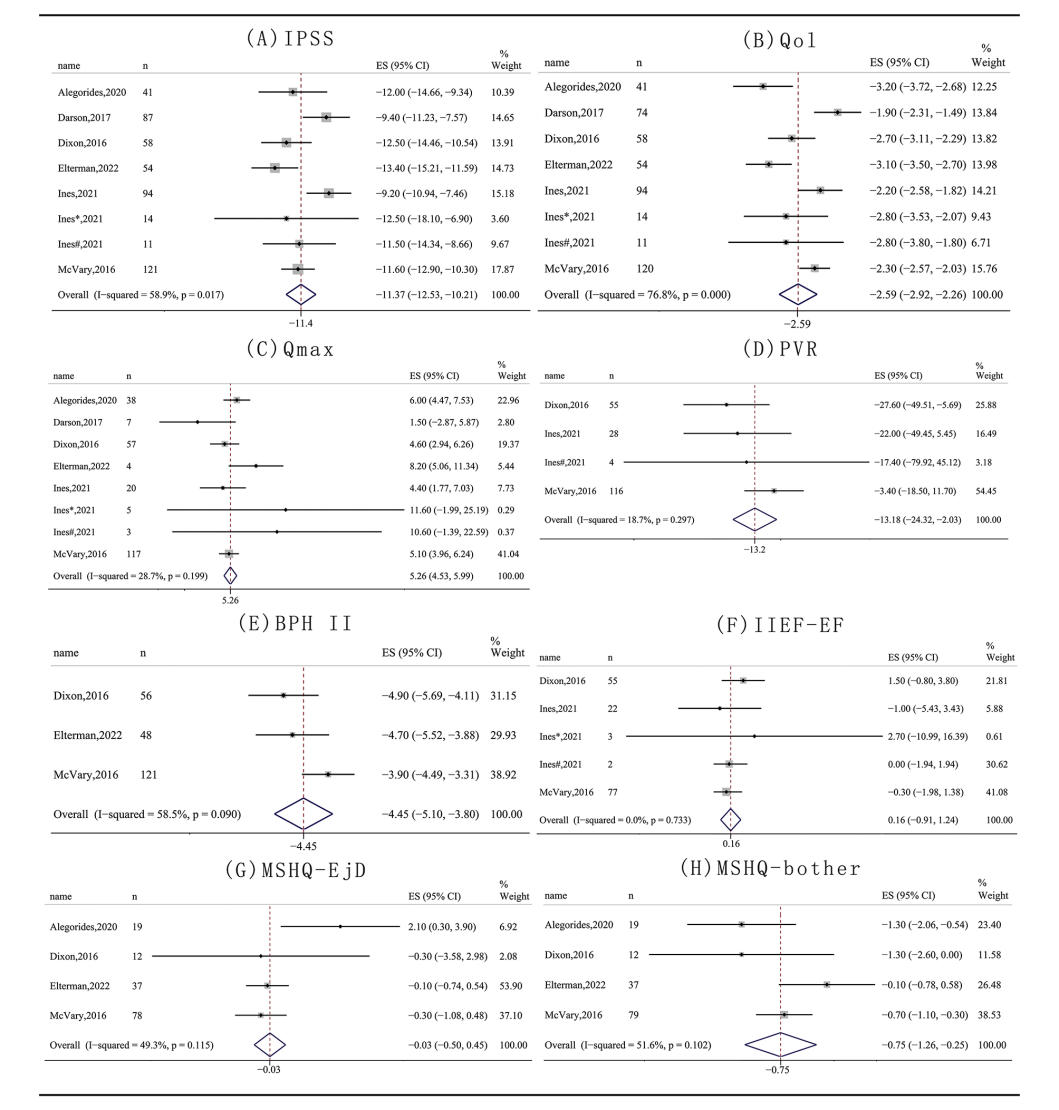
Forest plots of IPSS (**A**), Qol (**B**), Qmax (**C**), PVR (**D**), BPH II (**E**), IIEF-EF (**F**), MSHQ-EjD (**G**), MSHQ-bother (**H**) at one-year follow-up *Abbreviations: mos: months; IPSS: International Prostate Symptom Score; Qol: IPSS quality of life scale; PVR: postvoid residual; Qmax: maximum urine flow rate*

All results are summarized in Table 4, and the forest plots are shown in Fig. 2 and Additional File. In primary outcomes, IPSS, Qol and Qmax were significantly improved at six months after WVTT. At one year postoperatively, IPSS decreased by 11.37 (95% CI: -12.53, -10.21, p < 0.001, I²=58.9%); Qol decreased by 2.59 (95% CI: -2.92, -2.26, p < 0.001, I²=67.8%); and Qmax increased by 5.26 ml/s (95% CI: 4.53, 5.99, p < 0.001, I²=28.7%).

**Table 4:** Pooled outcomes at 1, 3, 6, and 12 months postoperatively and complication incidence rates

Outcomes	NO. of patients	Pooled effect	95%CI	P	I^2^	Exclude
IPSS 1mo	635	-6.77	(-8.24, -5.30)	< 0.001	77.50%	
IPSS 3mos	502	-11.26	(-12.52, -9.99)	< 0.001	63.40%	
IPSS 6mos	461	-12.20	(-13.00, -11.41)	< 0.001	19.80%	
IPSS 12mos	480	-11.37	(-12.53, -10.21)	< 0.001	58.90%	
Qol 1mo	612	-1.41	(-1.80, -1.03)	< 0.001	83.60%	
Qol 3mos	489	-2.35	(-2.65, -2.04)	< 0.001	67.90%	
Pre-Qol 6mos	448	-2.61	(-2.96, -2.25)	< 0.001	74.6%	
Qol 6mos	407	-2.45	(-2.62, -2.27)	< 0.001	35.60%	[11]
Qol 12mos	466	-2.59	(-2.92, -2.26)	< 0.001	76.80%	
Qmax 1mo	344	2.74	(1.83, 3.66)	< 0.001	56.90%	
Pre-Qmax 3mos	314	4.54	(2.6, 6.47)	< 0.001	89.60%	[8]
Qmax 3mos	312	5.28	(4.54, 6.01)	< 0.001	44.30%	
Qmax 6mos	280	5.15	(4.45, 5.85)	< 0.001	0	
Qmax 12mos	251	5.26	(4.53, 5.99)	< 0.001	28.70%	
PVR 3mos	231	-12.42	(-19.88, -4.96)	< 0.001	28.60%	[2, 4, 11]
PVR 6mos	212	-9.48	(-19.72, 0.75)	0.137	20.10%	
PVR 12mos	203	-13.18	(-24.32, -2.03)	< 0.001	18.70%	
BPH II 3mos	293	-3.74	(-4.60, -2.89)	< 0.001	76.80%	
BPH II 6mos	263	-4.45	(-4.83, -4.07)	< 0.001	48.20%	
BPH II 12mos	225	-4.45	(-5.10, -3.80)	< 0.001	58.50%	
Pre-IIEF-EF 6mos	179	-0.05	(-1.17, 1.67)	0.953	74.50%	
IIEF-EF 6mos	175	0.37	(-0.71, 1.45)	0.439	15.90%	[8]
IIEF-EF 12mos	159	0.16	(-0.91, 1.24)	0.768	0	
MSHQ-EjD 1mo	90	0.31	(-0.53, 1.16)	0.936	34.10%	
MSHQ-EjD 3mos	189	0.34	(-0.26, 0.94)	0.264	0	
Pre-MSHQ-EjD 6mos	161	0.89	(-0.51, 2.29)	0.213	70.80%	
MSHQ-EjD 6mos	149	0.09	(-0.55, 0.74)	0.699	49.30%	[11]
MSHQ-EjD 12mos	146	-0.03	(-0.50, 0.96)	0.728	49.30%	
MSHQ-bother 1mo	90	-0.35	(-0.67, -0.03)	0.076	26%	
MSHQ-bother 3mos	189	-0.36	(-0.62, -0.11)	0.027	45.10%	
MSHQ-bother 6mos	161	-0.66	(-1.23, -0.10)	0.022	66.40%	
MSHQ-bother 12mos	147	-0.75	(-1.26, -0.25)	0.004	51.60%	
Adverse events						
Pre-Hematuria	582	22%	(8%, 42%)	< 0.01	96.41%	
Hematuria	339	14%	(10%, 18%)	< 0.01	0	[6, 8]
Dysuria	112	21%	(14%, 29%)	< 0.01	77.38%	
Hematospermia	42	10%	(2%, 22%)	< 0.01	89.47%	
UTI	544	7%	(3%, 11%)	< 0.01	68.38%	
Urinary retention	741	12%	(6%, 19%)	< 0.01	86.07%	
Urinary urgency	414	10%	(5%, 17%)	< 0.01	73.28%	
Urinary frequency	338	10%	(3%, 20%)	< 0.01	85.78%	
Pelvic pain	335	3%	(1%, 6%)	< 0.01	0	
Retreatment	764	3%	(2%, 5%)	< 0.01	0	

Abbreviations: *mos: months; IPSS: International Prostate Symptom Score; Qol: IPSS quality of life scale; PVR: postvoid residual; Qmax: maximum urine flow rate; BPH II: Benign Prostate Hypertrophy Impact Index; IIEF-EF: International Index of Erectile Function erectile function domain; MSHQ-EjD: Male Sexual Health Questionnaire for Ejaculatory Dysfunction; UTI: urinary tract infection; CI: confidence intervals*

When analysing the results of PVR, we found significant heterogeneity in all results. We found that some patients in the studies by Alegorides et al. [11] (8/62 12.9%), Darson et al. [2] (3/131 2.3%), and Elterman et al. [4] (39/229 17%) had a history of urinary retention, which may introduce a bias in the effect measure of PVR. Excluding these studies resulted in a significant reduction in heterogeneity and a more stable pooled effect size. After excluding these studies, PVR significantly decreased by 13.18 mL (95% CI: -24.32, -2.03, p < 0.001, I²=18.7%) at one year postoperatively.

In the secondary outcomes, both BPH II and MSHQ-bother improved significantly at 6 months postoperatively and remained significant at one year postoperatively. BPH II was reduced by 4.45 (95% CI: -5.1, -3.8, p < 0.001, I²=58.5%), and MSHQ-bother was reduced by 0.75 (95% CI: -1.26, − 0.25, p = 0.004, I²=51.6%) at one year postoperatively. In contrast, WVTT did not significantly improve IIEF-EF and MSHQ-EjD. IIEF-EF was increased by 0.16 (95% CI: -0.91, 1.24, p = 0.768, I²=0), and MSHQ-EjD was decreased by 0.03 (95% CI: -0.5, 0.96, p = 0.728, I²= 49.3%) at one year postoperatively.

### Safety outcomes

The most commonly reported postoperative complications of WVTT included hematuria, dysuria, hematospermia, UTI, urinary retention, urinary urgency, urinary frequency and pelvic pain. The proportional analysis of these adverse events with 95% CIs is summarized in Table 4, and relevant forest plots are shown in Additional File. The most common adverse events were dysuria (21%, 95% CI: 14%, 29%, p < 0.01, I²=77.38%) and hematuria (14%, 95% CI: 10%, 18%, p < 0.01, I²=0). Two studies reported a high incidence of hematuria [6, 8]. The definition of hematuria may contribute to the high incidence of hematuria. After excluding these studies, the interstudy heterogeneity was significantly reduced, resulting in a more stable result. Moreover, the pooled retreatment rate after WVTT was 3% (95% CI: 2%, 5%, p < 0.01, I²=0) (Additional File).

### Sensitivity analysis and publication bias

We identified unstable studies in the sensitivity analysis of Qol, Qmax, IIEF-EF, and MSHQ-EjD, and the results before and after excluding the unstable studies are summarized in Table 4. In addition, we performed Egger’s tests in studies that reported changes in IPSS, Qol, Qmax and PVR, and no significant publication bias was found (IPSS: p = 0.76, Qol: p = 0.32, Qmax: p = 0.57, PVR = 0.492).

## Discussion

Currently, 30–50% of men over 50 years and 80% of men over 80 years suffer from moderate to severe LUTS due to BPH [17]. TURP is still considered the gold standard medical treatment but is associated with numerous adverse events [18]. Some vaporization of the prostate by laser or plasma vaporization has been shown to have no significant differences in treatment outcomes and postoperative complication rates [19].

Water vapor thermal therapy (WVTT) by the Rezūm^®^ system is a recently developed MIST that employs heated steam injected into the prostatic transition zone to thermally ablate hyperplastic tissue while localizing the treatment to specific locations [20]. This is an outpatient procedure with low anaesthesia requirements that effectively relieves LUTS while minimizing sexual side effects, reducing the risk of long-term hospitalization and other more invasive procedures [21].

The purpose of our study was to discuss the efficacy and safety of WVTT in the treatment of BPH with a one-year follow-up. We found that WVTT had a significant improvement in LUTS that persisted through the one-year follow-up period and had no apparent effect on sexual function.

Our study found that IPSS, Qol and Qmax changed significantly after surgery and maintained their efficacy during the one-year follow-up period. Within one year, IPSS decreased by 11.37, Qol decreased by 2.59, and Qmax increased by 5.26 ml/s. This is similar to the results of the randomized controlled trial conducted by Mcvary et al. [22] In fact, these results changed significantly in the first month postoperatively, and in the third month, the effect was increased by 66.3%, 66.7%, and 92.7%, respectively, compared with the first month. However, at six months and one year postoperatively, the effect was only maintained and not further. Compared to the effect at six months postoperatively, the symptomatic improvement in IPSS decreased by only 6.8%, Qol increased by only 5.7%, and Qmax increased by only 2.1%. However, according to the American Urological Association, patients with a decrease in IPSS of more than 3 points from baseline were considered responders. Therefore, the efficacy of WVTT on IPSS was significant. BPH II also maintained efficacy with a significant reduction of 4.45 at one year postoperatively.

Given the heterogeneity resulting from studies that included patients with a history of urinary retention, we included only the remaining studies for analysis and found that PVR decreased significantly by 13.2 ml at the one-year follow-up. However, after excluding these studies, the improvement in PVR at six months postoperatively became nonsignificant. This is because the measurement of PVR may be influenced by several factors, such as patient history and measurement method. Therefore, more studies are needed to standardize measurements in patients with the same disease characteristics to accurately assess the efficacy of WVTT on PVR. Unfortunately, no other literature has reported the efficacy of WVTT at longer follow-ups. Only Dixon et al. [3] and Mcvary et al. [22] reported that IPSS, Qol, Qmax, PVR and BPH II were still effective two years postoperatively. We look forwards to more studies with longer follow-up to explore the efficacy of WVTT over a longer period.

Regarding the effects on sexual function, the changes in IIEF-EF and MSHQ-EjD were not significant. For MSHQ-bother, there was a significant decrease of 0.75 at one year postoperatively. In contrast to the possible risk of retrograde ejaculation after TURP [23], no de novo erectile dysfunction or ejaculatory dysfunction was reported in our studies. A randomized controlled study conducted by McVary et al. reported the preservation of sexual function in BPH patients at five years by WVTT [24]. From this, we can infer that WVTT has no significant effect on the patient’s erection and ejaculation function and can also improve the patient’s sexual life bother to a certain extent. Cocci et al. reviewed the Rezum procedure and likewise reported its ability to preserve sexual function [25]. This may make young and sexually active BPH patients more inclined to choose it.

In our study of safety outcomes, we found that the most common adverse events included dysuria (21%), hematuria (14%), urinary retention (12%), hematospermia (10%), urinary frequency (10%) and urinary urgency (10%). We obtained these stable results after excluding some studies with large differences in patient baselines that had a large impact on the pooled outcomes. Most of these adverse events occurred within three months postoperatively and were resolved within three weeks. Only Dixon et al. [3], Ines et al. [8], and Mcvary et al. [7] reported 1 (1.5%), 3 (2.1%), and 2 (1.5%) patients with surgery-related serious AEs (> Clavien II), respectively. Most importantly, the postoperative retreatment rate of WVTT is only 3%, which has obvious advantages compared to TURP (8%) [23].

In fact, there is also current concern about the suitability of WVTT in patients with middle lobe obstruction, large prostate (> 80 ml), and indwelling catheter. In our study, we did not limit the population. However, the final results still showed the efficacy of WVTT. Barber et al. [26]. and Elterman [27]et al. have reported the efficacy of WVTT in patients with middle lobe obstruction and a large prostate. A systematic review reported the applicability of WVTT in patients with catheters secondary to BPH, especially in frail patients with comorbidities who could not receive general anaesthesia [28]. Another prospective study of 24 patients considered unsuitable for conventional BPH surgery or at high risk of complications reported that all patients had their indwelling catheters removed and returned to spontaneous urination during the one-year follow-up period [29]. Therefore, the efficacy of WVTT in such complex cases is also promising. As an outpatient procedure, WVTT has unique advantages. Alegorides et al. [11], Elterman et al. [4], and Wasserbauer et al. [5] all reported the average operative time as 6 min, 4.8 min, and 6.2 min, respectively, which was more advantageous than the average TURP operation of 19.64 min [30]. Moreover, WVTT requires only a simple form of anaesthesia, including intravenous sedation, oral sedation, prostate block, etc. Three studies reported that the patients were discharged on the same day [5, 6, 11]. Sahakyan et al. [31]. reported by cost-utility analysis that WVTT could achieve higher efficacy at a lower cost. Therefore, WVTT can be conducted under a simpler anaesthesia method without the need for ventilator auxiliary support and bladder irrigation, which can reduce medical financial and nursing pressure and the economic burden on patients. This seems to be especially important in the COVID-19 era.

Our study has several limitations. First, this is a single-arm meta-analysis that did not compare the differences between WVTT and other treatment modalities for BPH. Second, this meta-analysis included a small number of studies with small sample sizes. Third, some studies were not included in our meta-analysis according to the article screening criteria, which may introduce bias. Fourth, only one RCT was included in this analysis, and the remaining studies were single-arm trials and lacked controls. Fifth, the baseline characteristics of the included patients were inconsistent, resulting in strong heterogeneity in some results, but we could not perform subgroup analysis due to the lack of relevant studies and subgroup data.

## Conclusion

WVTT can provide sustained and significant efficacy in the treatment of BPH. For men with BPH, WVTT is a potential first-line therapeutic strategy compared to medications or more invasive surgical procedures.

## Electronic supplementary material

Below is the link to the electronic supplementary material.

Additional File: Efficacy and Safety of Water Vapor Thermal Therapy in the Treatment of Benign Prostate Hyperplasia: a Systematic Review and Single-arm Meta-analysis

## Data Availability

All data generated or analysed during this study are included in this published article.
