# Supplementary material for: Efficacy and safety of Water Vapor Thermal Therapy in the treatment of benign prostate hyperplasia: a systematic review and single-arm Meta-analysis
Source: BMC Urol. 2023 Apr 28;23:72. doi: 10.1186/s12894-023-01237-2 (PMC10147364; doi:10.1186/s12894-023-01237-2)
Supplement: Supplementary file 1 — Additional File: Efficacy and Safety of Water Vapor Thermal Therapy in the Treatment of Benign Prostate Hyperplasia: a Systematic Review and Single-arm Meta-analysis [file 12894_2023_1237_MOESM1_ESM.docx]

**Supplementary Materials**

**Efficacy and Safety of Water Vapor Thermal Therapy in the Treatment of Benign Prostate Hyperplasia: a Systematic Review and Single-arm Meta-analysis**

Junyi Yang ^a,1^, Weisong Wu ^a,1^, Yirixiatijiang Amier ^a^, Xianmiao Li ^a^, MMed Wenlong Wan ^a^, Chang Liu ^b^, Yucong Zhang ^b,*^, Xiao Yu ^a,*^

^a^ Department of Urology, Institute of Urology, Tongji Hospital, Tongji Medical College, Huazhong University of Science and Technology, Wuhan 430030, China

^b^ Department of Geriatrics, Tongji Hospital, Tongji Medical College, Huazhong University of Science and Technology, Wuhan 430030, China

**Contents**

(1) **Supplementary Fig. 1** Forest plots of IPSS (A), Qol (B), Qmax (C), PVR (D), BPH II (E), IIEF-EF (F), MSHQ-EjD (G), MSHQ-bother (H) at six-months follow-up

(2) **Supplementary Fig. 2** Forest plots of the incidence rate of dysuia (A), hematuria (B), urinary retention (C), hematospermia (D), urinary urgency (E), urinary frequency (F), urinary tract infection (G) and pelvic pain (H)

(3) **Supplementary Fig. 3** Forest plot of retreatment rate after WVTT

(4) **Supplementary Table 1** Search Strategy


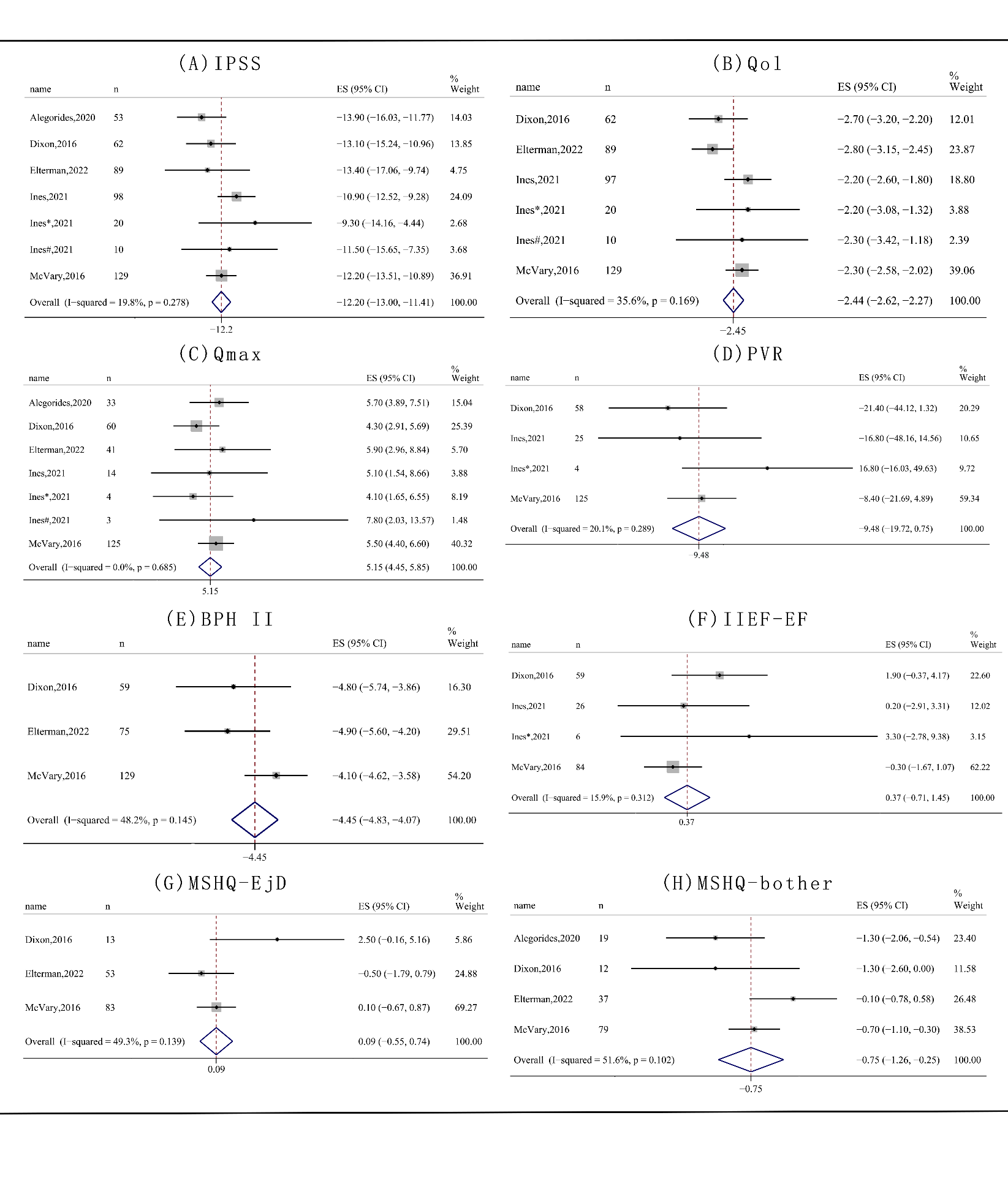
**Supplementary Fig. 1:** Forest plots of IPSS (A), Qol (B), Qmax (C), PVR (D), BPH II (E), IIEF-EF (F), MSHQ-EjD (G), MSHQ-bother (H) at six-months follow-up. Abbreviations: *mos: months; IPSS: International Prostate Symptom Score; Qol: IPSS quality of life scale; PVR: post-void residual; Qmax: maximum urine flow rate.*

**Supplementary Fig. 2:** Forest plots of the incidence rate of dysuia (A), hematuria (B), urinary retention (C), hematospermia (D), urinary urgency (E), urinary frequency (F), urinary tract infection (G) and pelvic pain (H).


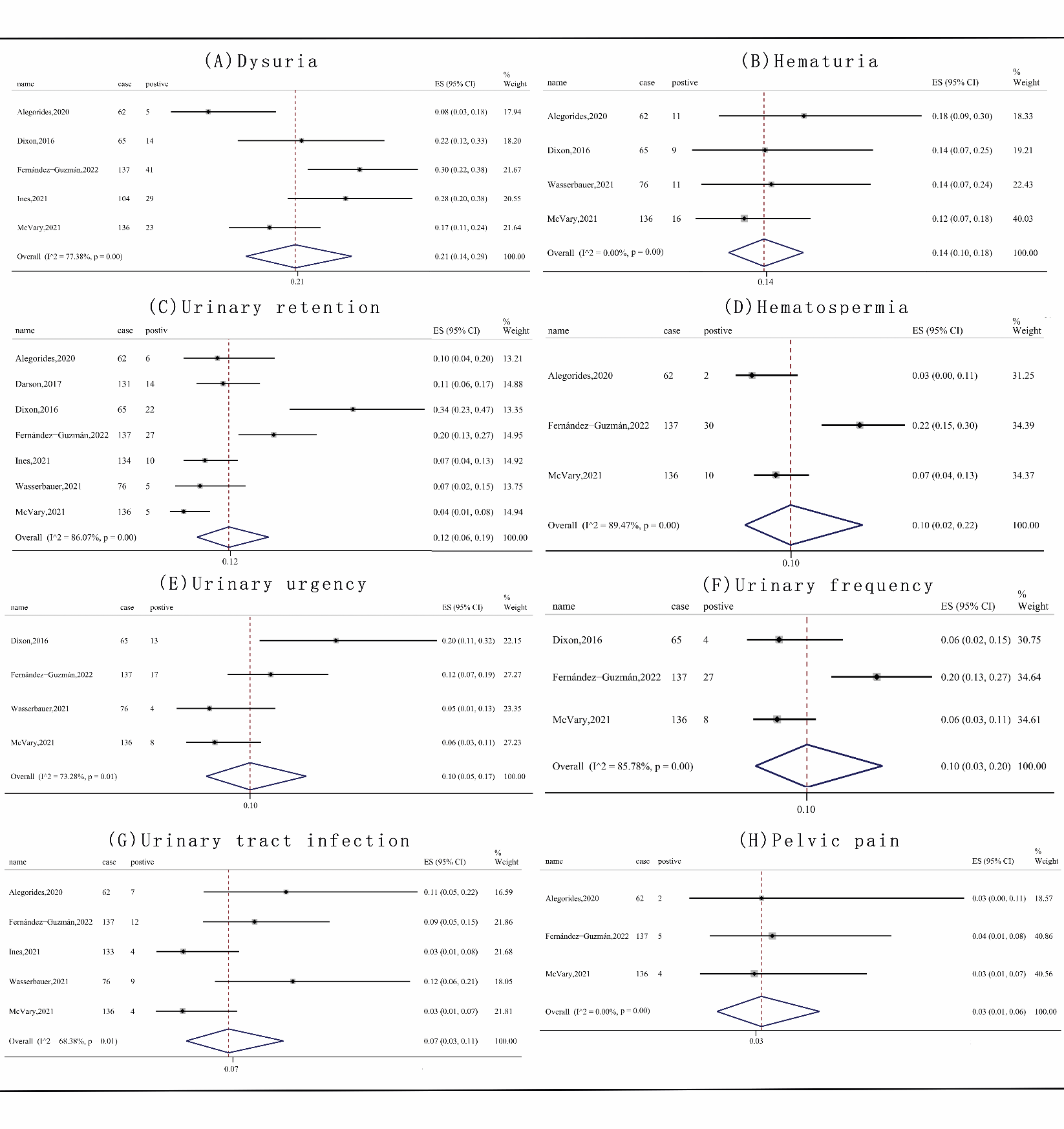


**Supplementary Fig. 3:** Forest plot of retreatment rate after WVTT.


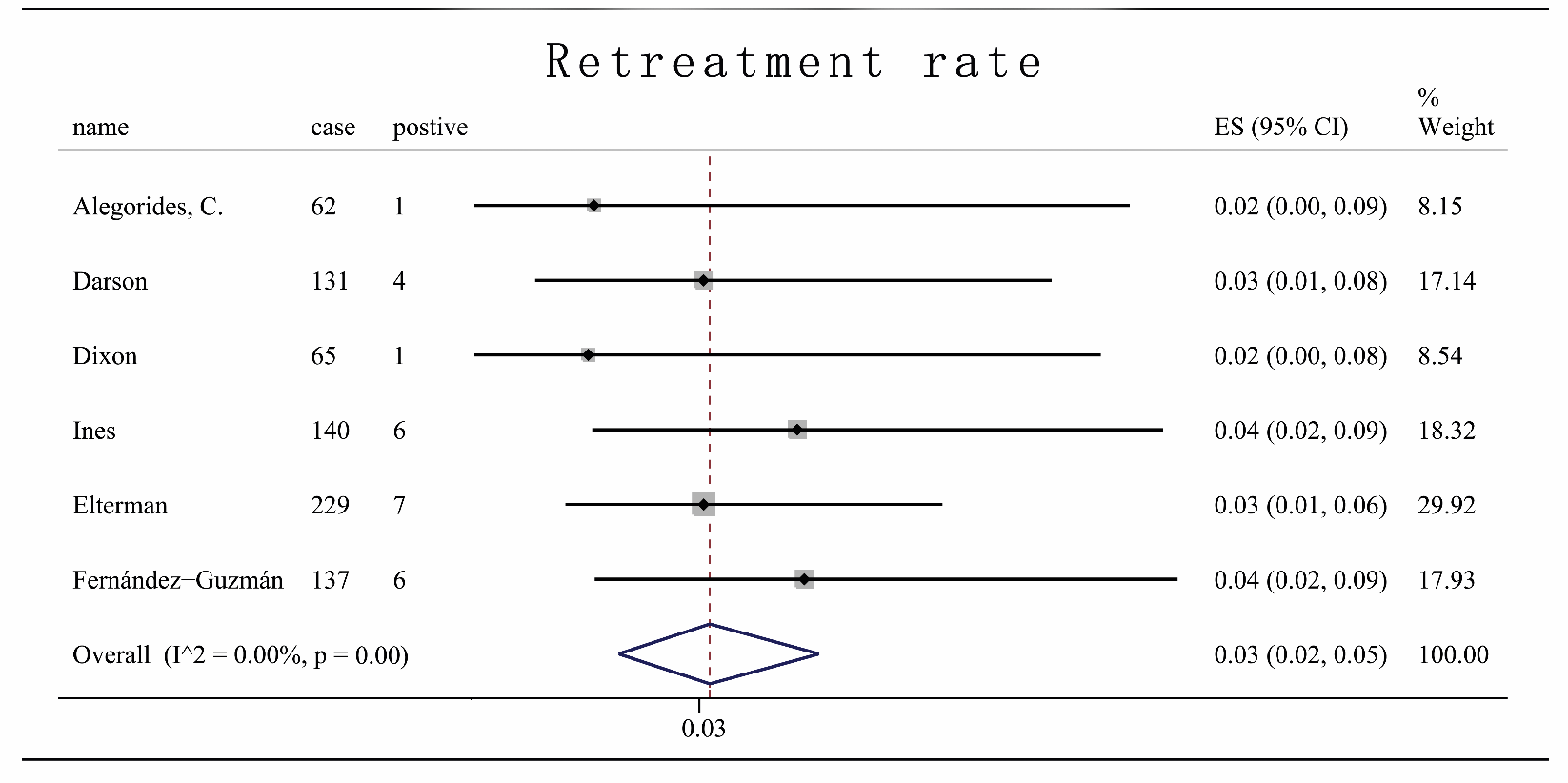


**Supplementary Table 1:** Search Strategy

**PubMed**

**Number of localized studies:** **138**

|  | **Descriptors** | Number of studies reached |
| --- | --- | --- |
| **#1** | "Prostatic Hyperplasia"[MeSH Terms] OR "BPH"[Title/Abstract] OR "benigh prostatic hyperplasia"[Title/Abstract] OR "prostatic adenoma"[Title/Abstract] OR "benign prostatic hypertrophy"[Title/Abstract] OR "prostatic hypertrophy"[Title/Abstract] | 29923 |
| **#2** | "water vapour thermal therapy"[Title/Abstract] OR "WVTT"[Title/Abstract] OR "Rezum"[Title/Abstract] | 150 |
| **#3** | **#1** AND **#2** | 138 |

**Web Of Science**

**Number of localized studies: 208**

|  | **Descriptors** | Number of studies reached |
| --- | --- | --- |
| **#1** | TS=(Prostatic Hyperplasia OR BPH OR Benigh Prostatic Hyperplasia OR Prostatic Adenoma OR Benign Prostatic Hypertrophy OR Prostatic Hypertrophy) | 49780 |
| **#2** | TS=(water vapour thermal therapy OR WVTT OR Rezum) | 488 |
| **#3** | **#1** AND **#2** | 208 |

**Embase**

**Number of localized studies: 6**

|  | **Descriptors** | Number of studies reached |
| --- | --- | --- |
| **#1** | Prostatic Hyperplasia:ab,ti OR BPH:ab,ti OR Benigh Prostatic Hyperplasia:ab,ti OR Prostatic Adenoma:ab,ti OR Benign Prostatic Hypertrophy:ab,ti OR Prostatic Hypertrophy:ab,ti | 3895 |
| **#2** | water vapour thermal therapy:ab,ti OR WVTT:ab,ti OR Rezum:ab,ti | 283 |
| **#3** | **#1** AND **#2** | 6 |

**Cochrane Library**

**Number of localized studies:** 48

**Limited: Trails**

|  | **Descriptors** | Number of studies reached |
| --- | --- | --- |
| **#1** | (Prostatic Hyperplasia):ab,ti,kw OR (BPH):ab,ti,kw OR (Benigh Prostatic Hyperplasia):ab,ti,kw OR (Prostatic Adenoma):ab,ti,kw OR (Benign Prostatic Hypertrophy):ab,ti,kw OR (Prostatic Hypertrophy):ab,ti,kw | 4051 |
| **#2** | (water vapour thermal therapy):ab,ti,kw OR (WVTT):ab,ti,kw OR (Rezum):ab,ti,kw | 60 |
| **#3** | **#1** AND **#2** | 49 |
